# A Case of Neonatal Cytotoxic Lesions of the Corpus Callosum With Subpial Hemorrhage

**DOI:** 10.7759/cureus.77636

**Published:** 2025-01-18

**Authors:** Yoshihito Ban, Hiroshi Tagawa, Kai Wakatsuki, Ryutaro Onishi, Toshiyuki Noda, Masami Sugawara, Kumiko Ando

**Affiliations:** 1 Diagnostic Radiology, Kobe City Medical Center General Hospital, Kobe, JPN; 2 Pediatrics and Neonatology, Kobe City Medical Center General Hospital, Kobe, JPN

**Keywords:** cytotoxic lesions of the corpus callosum, magnetic resonance imaging, pediatrics and neonatology, radiology, splenium of the corpus callosum, subpial hemorrhage

## Abstract

Subpial hemorrhage is a rare type of birth-related brain hemorrhage in neonates that can manifest with symptoms such as apnea and seizures. On brain magnetic resonance imaging (MRI), subpial and parenchymal hemorrhages are typically observed but often resolve without lasting sequelae. Cytotoxic lesions of the corpus callosum (CLOCCs) are not as common in neonates as they are in adults and children. We present a rare case of a term neonate with subpial hemorrhage accompanied by CLOCCs who experienced a favorable clinical course.

A full-term female infant was delivered via emergency cesarean section. At 18 hours post-birth, the infant experienced recurrent seizures. Following phenobarbital administration and the initiation of respiratory support, a brain MRI performed on day 6 revealed hematomas in the subpial and subcortical regions, leading to a diagnosis of subpial hemorrhage. Additionally, diffusion restriction was noted in the corpus callosum and optic radiation extending to the posterior limb. Follow-up MRI on day 14 of life showed the resolution of most of the lesions, confirming the diagnosis of CLOCCs. The infant was discharged on day 22 without further seizures and has shown normal growth and development for 18 months post-discharge.

Thus, this case reports a neonatal patient with typical radiological features and clinical progression of subpial hemorrhage and CLOCCs, leading to a favorable outcome. The positive outcome underscores the importance of understanding the unique radiological features and progression of these conditions. In neonates presenting with splenial lesions associated with brain hemorrhage, it is crucial to monitor the resolution of symptoms and lesions to rule out alternative diagnoses. Furthermore, continued developmental monitoring is recommended to ensure optimal outcomes.

## Introduction

Subpial hemorrhage represents a rare form of intracranial hemorrhage observed in term neonates without prenatal abnormalities, trauma, or complications or high Apgar scores [1]. It is characterized by bleeding in the subpial space, a potential space between the pia mater and the glial layer of the cortex. It may also be accompanied by cortical infarction and parenchymal hemorrhage. In affected term neonates, apnea and seizures are common clinical manifestations, which typically respond well to anticonvulsant therapy. Despite involving damage to the brain parenchyma, in most cases, hematomas are absorbed over time without surgical drainage, generally resolving without long-term sequelae [2,3].

Cytotoxic lesions of the corpus callosum (CLOCCs), also known as mild encephalopathy with reversible splenial lesions or reversible splenial lesion syndrome, are observed as a reversible diffusion restriction in the splenium of the corpus callosum on magnetic resonance imaging (MRI). The lesion is associated with various etiologies, including bacterial and viral infections, metabolic disorders, drugs, epilepsy, malignancies, and brain hemorrhages [4,5]. CLOCCs are characterized by cytotoxic edema as their underlying mechanism and are recognized as secondary lesions. Typical symptoms in patients with CLOCCs commonly include seizures, impaired consciousness, and delirium [6]. Radiological findings include high signal intensity on T2-weighted imaging and fluid-attenuated inversion recovery, low signal intensity on T1-weighted imaging and during the acute phase, high signal intensity on diffusion-weighted image (DWI), and decreased apparent diffusion coefficient (ADC) values [6]. Lesions are categorized into three patterns: a small round or oval lesion located in the center of the splenium, a lesion centered in the splenium but extending through the callosal fibers laterally into the adjacent white matter, or a lesion centered posteriorly but extending into the anterior portion of the corpus callosum [7]. Prognostically, CLOCCs are associated with generally favorable clinical and radiological outcomes. Lesions typically disappear within a week on imaging and clinical symptoms complete recovery without sequelae [5].

Though CLOCCs are well-documented in adults and children, reports in neonates, particularly those associated with brain hemorrhage, are uncommon. In neonates, similar transient high signal intensity in the splenium of the corpus callosum on DWI is seen with hypoxic-ischemic encephalopathy (HIE), intraparenchymal hemorrhage, ischemia, stroke, and brain abscess and may be associated with poor prognosis [8,9]. This case report highlights a rare occurrence of CLOCCs with subpial hemorrhage in a full-term neonate with a good clinical course.

## Case presentation

A full-term female infant was delivered via emergency cesarean section at 36 weeks and five days due to labor arrest and fetal distress. The mother had a history including pelvic fracture and vaginal laceration due to trauma, with significant pelvic adhesions and bladder injury during cesarean section. The neonate's birth weight was 2726 g, and her Apgar scores were 2, 5, and 8 points at one, five, and 10 minutes, respectively, indicating neonatal asphyxia. A subcutaneous mass was observed in the left parietal region, and the infant required respiratory support due to weak respiratory effort and was admitted to the neonatal intensive care unit. Blood tests revealed hypoglycemia, which was promptly corrected with intravenous glucose administration. Respiratory support was discontinued following stabilization.

**Figure 1:**
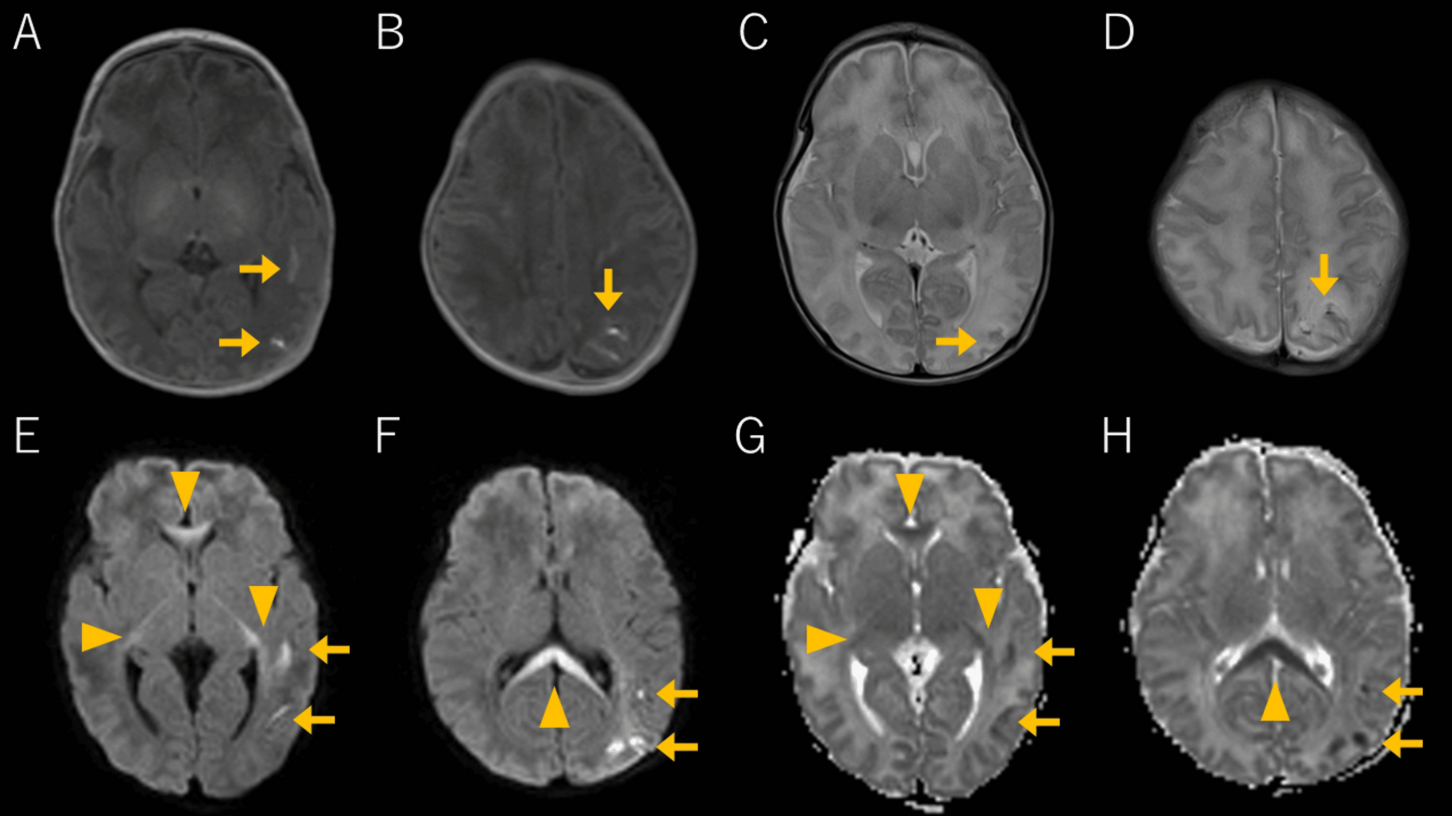
Brain MRI findings on day 6 Subpial hemorrhages are indicated by arrows (→), whereas CLOCCs are marked with arrowheads (▲). Axial T1-weighted images show subpial hemorrhage and cortical-subcortical hematomas with high signal intensity in the left temporoparietal region (A, B). Axial T2-weighted images show high signals in the cerebral cortex and low signal hematomas (C, D). DWI reveals high-signal-intensity lesions in the cortex below the subpial hematoma and reveals the corpus callosum and optic radiation to the posterior limb (E, F). The mean ADC values of the splenium were 663×10^-6^ mm^2^/s (G, H). MRI: magnetic resonance imaging; CLOCCs: cytotoxic lesions of the corpus callosum; DWI: diffusion-weighted imaging; ADC: apparent diffusion coefficient

**Figure 2:**
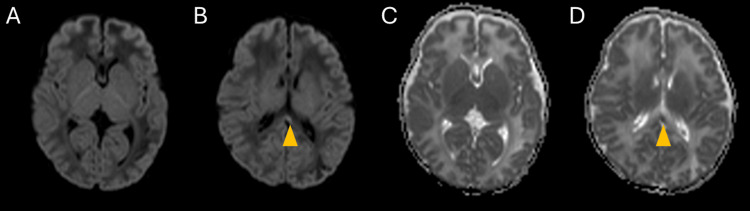
MRI findings on day 14 On DWI, diffusion restriction is mostly resolved (A, B). A part of the lesions that remained in the splenium of the corpus callosum are marked with arrowheads (▲). The mean ADC values of the splenium were 1,298×10^-6^ mm^2^/s (C, D). MRI: magnetic resonance imaging; DWI: diffusion-weighted imaging; ADC: apparent diffusion coefficient

At 18 hours after birth, the infant experienced recurrent seizures involving the right upper limb and both lower limbs, accompanied by apnea. Phenobarbital was administered, and respiratory support was re-initiated. The electroencephalogram (EEG) showed high-amplitude slow waves, but other tests, including blood tests, chest radiography, and cranial, cardiac, and abdominal ultrasounds, yielded normal results. The seizures resolved by day 2, allowing for the discontinuation of phenobarbital and respiratory support on day 4. Brain MRI performed on the sixth day after birth revealed hematomas with high signal intensity on T1-weighted images and low signal intensity on T2-weighted images, predominantly in the subpial and subcortical regions, mainly in the left parietal lobe (Figure 1). Additionally, DWI and ADC maps revealed restricted diffusion in the cortex beneath the hematoma, leading to a diagnosis of subpial hemorrhage. Restricted diffusion was also observed in the corpus callosum and the optic radiation to the posterior limb of the internal capsule (the mean ADC values of the splenium were 663×10^-6^ mm^2^/s). Follow-up MRI on day 14 revealed substantial resolution of these lesions, leading to a diagnosis of CLOCCs (Figure 2; the mean ADC values of the splenium were 1,298×10^-6^ mm^2^/s). No abnormal signals were detected in the basal ganglia or brainstem during either MRI scans, ruling out HIE. The infant was discharged on day 22 without further seizures and has demonstrated normal growth and development for 18 months post-discharge.

## Discussion

Subpial hemorrhage is a rare type of intracranial hemorrhage in neonates, typically resolving without long-term sequelae. However, fatal outcomes have been reported in preterm infants, indicating a higher mortality risk in non-term infants [2,3]. One hypothesis for the mechanism of subpial hemorrhage is the intense pressure and forces during delivery, which may compress fragile subpial vessels, causing rupture and hemorrhage in the superficial brain parenchyma and leptomeninges. Scalp hematoma has been reported in 70.6% of cases with subpial hemorrhage [2]. MRI findings in subpial hemorrhage cases often show the characteristic "yin-yang sign" or "sandwich sign." Irrespective of the presence or degree of intraparenchymal hemorrhage, the consistent combination of a bright, hyperintense appearance of the cerebral cortex with the dark, hypointense overlying subpial hemorrhage on T2-weighted imaging and DWI creates a "yin-yang sign" [3]. The consistent presence of a bright, hyperintense subpial bleed, a dark thin fluid layer, and an underlying normal cortex on T2-weighted imaging and DWI resembles a "sandwich sign" [2].

Previous reports on CLOCCs in neonates predominantly involved full-term infants who presented with decreased responsiveness, poor feeding, and seizures. Coexisting conditions often include hypoglycemia, hyponatremia, and neonatal pneumonia; however, few reports have linked CLOCCs to cerebral hemorrhage [10,11]. Other than CLOCCs, corpus callosum lesions in neonates may also result from HIE and pre-Wallerian degeneration, conditions generally associated with poor prognoses.

In the present case, a full-term infant developed abnormalities observed on EEG, seizures, and apnea 18 hours after birth. The infant recovered uneventfully following treatment with anticonvulsants. On day 8, the MRI revealed subpial and subcortical hemorrhage and cortical infarction in the left temporoparietal lobe. The presence of prolonged labor and scalp hematoma in the infant's left parietal region suggested the risk of subpial hemorrhage. Additionally, diffusion restrictions observed in MRI, in the corpus callosum, optic radiation, and posterior limb of the internal capsule, improved by day 14, resulting in a diagnosis of CLOCCs. The splenium of the corpus callosum has strong connections with the temporo-occipital lobes, making it susceptible to damage from injuries to these lobes. It is thought that the subpial hemorrhage in the left parietal lobe caused spasms in the right upper extremity and both lower extremities and the cause of diffusion restriction in the corpus callosum appears to be associated with this hemorrhage and spasms. However, the exact cause and underlying mechanisms of the splenial lesion remain unclear. Possible contributing factors include perinatal asphyxia, hypoglycemia, and the use of anticonvulsant medications.

Several reports of CLOCCs associated with cerebral hemorrhage have been published in children and adults. Toi et al. identified corpus callosum lesions in 12.7% of patients with aneurysmal subarachnoid hemorrhage, the condition being more common in severe cases and younger patients but with no indications for poor prognosis [12]. Other reports have described the occurrence of CLOCCs after subarachnoid hemorrhage, arteriovenous malformation surgery, and postoperative chronic subdural hematoma surgery [13,14]. The proposed mechanism of corpus callosum lesions associated with cerebral hemorrhage is thought to involve elevated levels of interleukin (IL)-1, IL-6, and tumor necrosis factor-α in the cerebrospinal fluid (CSF). High levels of inflammatory cytokines lead to glutamate release from astrocytes and impaired glutamate reuptake, increasing the extracellular glutamate concentration and promoting water uptake into cells through aquaporins and the Na+-K+ pump, causing cellular edema [7]. In subpial hemorrhage, the hemorrhage and changes in the intracranial pressure may elevate CSF cytokine levels, triggering CLOCCs.

Similar splenial lesions have been observed in neonates with HIE. Lesions of the corpus callosum in HIE are found in 21.1% of patients treated with hypothermia and correlate with the incidence of seizures, intubation, mortality, and developmental delay at 12-18 months [15]. Although the mechanisms and reversibility of abnormal signals in the splenium in HIE remain unclear, the possibility of CLOCCs should be considered in differential diagnoses.

Pre-Wallerian degeneration associated with brain damage can also lead to diffusion restriction in the corpus callosum. Pre-Wallerian degeneration is an early change resulting from abnormalities in axons, which can manifest as Wallerian degeneration following cerebral hemisphere injury [8]. Follow-up over 30-90 days can reveal abnormalities in the myelin sheath. Additionally, pre-Wallerian degeneration serves as a prognostic indicator of persistent paralysis following stroke [8].

Although CLOCCs in neonates resolve within a week, some cases report developmental delays, particularly in language [16]. The corpus callosum plays a critical role in visuospatial information processing, vocabulary acquisition, reading, calculation, intelligence quotient, behavior, and consciousness [6]. Considering previous reports, neonatal corpus callosum lesions encompass various conditions, and non-CLOCCs can lead to long-term developmental impairment. We presented a rare case of a term neonate with subpial hemorrhage, which is generally associated with a good prognosis, complicated by CLOCCs, which also typically have a favorable prognosis. In this case, combining both conditions resulted in a favorable outcome. Therefore, when corpus callosum lesions are detected on neonatal MRI, it is crucial to confirm the reversibility of the lesions and monitor the course of the infant's growth and development.

## Conclusions

This case highlights a reversible lesion in the corpus callosum associated with subpial hemorrhage in a neonate, with a favorable outcome. Similar to older children and adults, neonates can develop CLOCCs as a result of brain hemorrhage. CLOCCs associated with subpial hemorrhage are distinct cerebral hemorrhage-related lesions of the corpus callosum and have a good prognosis, whereas HIE or pre-Wallerian degeneration should be carefully ruled out. When splenial lesions associated with brain hemorrhage are observed in neonates, it is crucial to monitor the resolution of symptoms and radiological findings.
